# A rapid review of evidence on the determinants of and strategies for COVID-19 vaccine acceptance in low- and middle-income countries

**DOI:** 10.7189/jogh.11.05027

**Published:** 2021-11-20

**Authors:** Sandeep Moola, Nachiket Gudi, Devaki Nambiar, Neha Dumka, Tarannum Ahmed, Isha Ramesh Sonawane, Atul Kotwal

**Affiliations:** 1 George Institute for Global Health, Hyderabad, India; 2 PATH, Bengaluru, India; 3 George Institute for Global Health, New Delhi, India; 4 The National Health Systems Resource Centre, New Delhi, India

## Abstract

**Background:**

Vaccine acceptance and hesitancy among the general population and health care workers play an important role in successfully controlling the Coronavirus Disease (COVID)-19 pandemic. While there is evidence for vaccine hesitancy across the globe, wide variation in factors influencing vaccine acceptance has been reported, mainly from High-Income Countries (HIC). However, the evidence from Low- and Middle-Income Countries (LMICs) remains unclear. The objective of this review was to describe the determinants of vaccine acceptance and strategies to address those in an LMIC context.

**Methods:**

The World Health Organization’s (WHO) Measuring Behavioral and Social Drivers of Vaccination (BeSD) Increasing Vaccination Model was employed to identify factors that influenced vaccine acceptance. All evidence related to supply-side and demand-side determinants and social and health system processes were examined. A comprehensive search for published literature was conducted in three databases and grey literature in relevant websites of government, multinational agencies, and COVID-19 resource aggregators, followed by a narrative synthesis.

**Results:**

Overall, the results showed that the vaccine acceptance rates differed across LMICs, with a wide variety of reasons cited for vaccine hesitancy. Vaccine acceptance was reportedly greater among males, those with higher education, elevated socio-economic status, the unmarried, those employed as health care workers. Evidence suggested that exposure to misinformation about COVID-19 vaccines and public concerns over the safety of vaccines may contribute to lower acceptance rates. Strategies to increase vaccine acceptance rates included direct engagement with communities through influencers, including community leaders and health experts; clear and transparent communication about COVID-19 vaccines, financial and non-financial incentives; and strong endorsement from health care workers. Trust in government was identified as a significant enabler of vaccine acceptance.

**Conclusions:**

There is a need for measures to address public acceptability, trust and concern over the safety and benefit of approved vaccines. Local context is essential to consider while developing programs to promote vaccine uptake. The governments worldwide also need to strategize to develop plans to address the anxiety and vaccine related concerns of community regarding vaccine hesitancy. There is a need for further research to evaluate strategies to address vaccine hesitancy in LMIC.

The COVID-19 pandemic has caused significant morbidity and mortality globally, with more than 3 million deaths reported worldwide as of May 2021 [1]. The situation in India has been grim, with 325 972 deaths reported so far [1]. Various virus mitigation measures have been in place; however, the risk of further outbreaks and disruption to societal and economic activities likely remains until effective vaccines are administered to prevent hospitalisation and limit infection and spread. Fast-track development of several novel vaccines has been under way to curb the spread of COVID-19 infection [2]. Backed by government support, the scientific community and pharmaceutical industry made huge strides towards developing efficacious and safe vaccines for COVID-19 [3]. However, vaccine hesitancy has been identified as a significant barrier towards achieving successful vaccine coverage [4].

The success of the COVID-19 vaccination programme will depend on the proportion of the population willing to be vaccinated, and recent estimates suggest that up to 70% of the population may require vaccination to bring an end to the current pandemic [5]. Thus, highlighting the need for a speedy rollout of vaccines to bring back normalcy as soon as possible. However, as the pandemic has evolved, several reports point towards public concern about the safety of COVID-19 vaccines due to their rapid development [6-8], widespread misinformation about COVID-19 vaccines [6,7,9], and distrust in governments [6-8], all of which may have influenced vaccine acceptance and uptake in populations worldwide. The latest data show that globally, as of 12 June 2021, 2.34 billion COVID-19 vaccine doses were administered. This included 1.6 billion people who received at least one dose of COVID-19 vaccine, and around 727 million fully vaccinated people (ie, received all doses as per the vaccination protocol) [10]. Reports suggest that 85% of the doses were administered in high- and upper-middle-income countries, compared to only 0.3% of doses administered in LMIC [11]. In India, for example, data updated on 22 June 2021 suggests that more than 280 million vaccine doses have been administered, with 51 million individuals having received both doses [12].

Vaccine hesitancy has been reported in a few surveys, although the prevalence of this phenomenon remains to be rigorously studied in LMIC [6-8,13,14]. It is important to understand factors that influence vaccine acceptance to inform measures to improve public acceptability and uptake of COVID-19 vaccines.

The Rapid Evidence Synthesis team at the George Institute for Global Health (TGI), India, and the Knowledge Management Division (KMD) of the National Health Systems Resource Centre (NHSRC), India, collaborated to identify evidence on; i) the determinants of vaccine acceptance and hesitancy, and ii) interventions that can promote vaccine acceptance. Additionally, the team reviewed relevant literature that contextualises evidence relevant to LMIC context.

## METHODS

**Figure 1:**
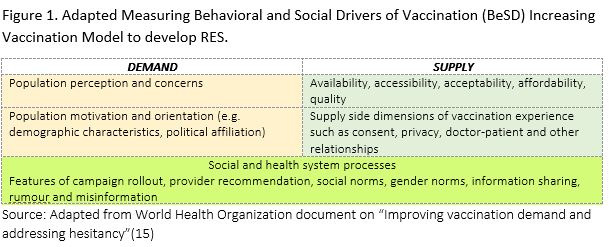
Adapted Measuring Behavioral and Social Drivers of Vaccination (BeSD) Increasing Vaccination Model to develop RES. Source: Adapted from World Health Organization document on “Improving vaccination demand and addressing hesitancy” [15].

An initial scoping of the literature was conducted. Following this, the World Health Organization’s (WHO’s) Measuring Behavioral and Social Drivers of Vaccination (BeSD) Increasing Vaccination Model (**Figure 1**) was utilized to identify influencing factors for vaccine acceptance [15]. Using this model, we examined evidence related to supply-side determinants (practical issues of availability, accessibility, affordability, cost, acceptability, and quality; and other vaccination-related factors at the systems level). Additionally, demand-side determinants (peoples’ perceptions such as risk perception and people's motivation, such as demographic characteristics), and social and health system processes were also assessed.

A comprehensive search of the published literature was conducted in PubMed, Health Systems Evidence and EMBASE databases and for grey literature in relevant websites of government, multinational agencies, and COVID-19 resource aggregators. The search protocol was developed based on the above framework and the objectives, with an emphasis on determinants, strategies, and the context. Relevant government advisories and policies, reports and guidelines related to vaccine acceptance and uptake were also searched. The searches were conducted on 28 and 29 April 2021 and were restricted to studies published in the English language, with no search date limits applied. We also screened references of included studies. Studies were screened for potential inclusion by reviewers (SM, NG, TA, IS) independently at the title and abstract screening stage in Rayyan database (Rayyan Systems Inc, Cambridge, Massachusetts), and at the full text screening stage. Disagreements on inclusion and exclusion of studies were resolved by consensus between the two reviewers or in consultation with a third reviewer. Multiple types and levels of evidence were considered to inform decision making to plan and develop resources for COVID-19 vaccine acceptance and uptake.

A pre-designed data extraction template was used, and a narrative synthesis was undertaken for the findings of the review. Data were extracted by an independent reviewer (NG, TA, IS) using a predesigned data extraction form and an experienced reviewer (SM, DN) assessed the correctness of the data by selecting 25% of the studies randomly. Relevant data on country/region, sample characteristics, study designs, determinants, strategies, and results were extracted. The evidence was structured and organised by sections corresponding to the study framework. Details on search strategies and a list of websites searched are provided in Appendix S1 and S2 in the **Online Supplementary Document**.

## RESULTS

A total of 15 documents were included in the final report following the study selection process based on the inclusion criteria [6-8,13,14]. The study selection process is presented in Appendix S3 in the **Online Supplementary Document** (Preferred Reporting Items for Systematic reviews and Meta-Analyses (PRISMA) flowchart). The records included two systematic reviews [8,14], one rapid review [16], six cross-sectional surveys [6,7,17-20], five reports [13,15,21-23], and one opinion piece [24]. Studies were conducted in Bangladesh, Brazil, India, Nigeria, Pakistan, South Africa, Zimbabwe, and Vietnam. As on 29 April 2021, ten studies were identified from a pre-print server (https://www.medrxiv.org/) relevant to the LMIC context, however, these were not included as these were not peer-reviewed and therefore, could not be used to inform or guide decision-making. However, a brief summary of the studies relevant to the LMIC context has been provided [25-34].

### Determinants of COVID-19 vaccine acceptance, and hesitancy

In a multinational survey, it was reported that the prevalence of COVID-19 vaccine acceptance in India was 74.5% based on a survey of 742 randomly sampled respondents from the general population [6]. In an opinion polling conducted in India, a high vaccine acceptance rate of 87% was reported in adults aged between 18 to 74 years [7]. These results indicated a relatively high rate of COVID-19 vaccine acceptance in India, which were similar to the rates reported in other lower-middle-income countries such as Brazil and South Africa [6]. Several reasons for COVID-19 vaccine hesitancy were also reported across studies. A summary of the key findings on the determinants of COVID-19 vaccine acceptance and hesitancy based on the three different domains of the BeSD model has been provided [15].

#### Demand-side determinants

Results indicated that population perception of COVID-19 vaccination's relative risks and benefits is a significant barrier to vaccine acceptance. However, among health care workers, greater susceptibility and severity of illness was associated with greater odds of vaccine acceptance [20].

Some of the included studies suggested that the COVID-19 vaccine hesitancy was associated with gender, attitudes, and the information source. Determinants such as gender (male), occupation (health care workers), and higher education significantly increased COVID-19 vaccine acceptance [17,18,20]. Overall, female respondents reported higher vaccine hesitancy compared to males, both in the general and health care worker population [17,18,20]. Higher education level (graduate and postgraduate level) and income resulted in increased COVID-19 vaccine acceptance [17,18]. In studies conducted in Bangladesh and Vietnam, being female, married, and lesser education had a significant negative impact on the participants’ intention to be vaccinated [17,20]. In a review from Pakistan, it was reported that religious beliefs played an important role in vaccine hesitancy [24].

In a study in Bangladesh, residents of slum, semi-urban, and rural areas were more vaccine-hesitant than those living in the cities [17]. Further, it was reported that almost 40% of the slum dwellers were hesitant to vaccinate against COVID-19. The same study reported that occupations such as agriculture, day-labour, and homemakers showed a low prevalence of vaccine acceptance [17]. Respondents who were divorced, separated, or widowed were found to be twice as likely to be vaccine-hesitant than single or unmarried. A study from Vietnam reported differences in COVID-19 vaccine acceptance across occupations [20]. Non-medical health care staff and those who received COVID-19 information from relatives had lower vaccine acceptance rates than doctors who did not receive information from their relatives [20]. Receiving information from relatives led to significant misconceptions and fears about vaccine safety [20]. Another study indicated that an individual’s political beliefs influence perceptions of the vaccine, its efficacy and safety [7].

#### Supply-side determinants

A study in Vietnam reported that the effectiveness and risk of severe side-effects of the COVID-19 vaccine contributed to vaccine hesitancy [20]. A Nigerian study found unreliability of clinical trials, high cost, and vaccine safety as the top three reasons for COVID-19 vaccine hesitancy [18]. A study from Bangladesh reported the importance of affordability. Almost three quarters of the participants were willing to get vaccinated against COVID-19 with a safe, effective and free vaccine, compared to 46.5% if there was a minimum fee [17]. Concerns about the safety of the vaccine were reported in several studies due to the rapid speed with which pharmaceutical companies developed COVID-19 vaccines [7,13,14,17,18,21]. In a survey conducted by the WHO, respondents expressed concerns about the risk of COVID-19 exposure when seeking vaccination [13].

Results from included studies indicated that a strong recommendation from a health care provider or an influential community member was a significant motivating factor to get vaccinated. In contrast, information from friends, family members, or social network contacts who choose not to get vaccinated decreased motivation for vaccination [7,8,15,18,20]. A survey in India found that men were more likely to accept their employer’s recommendation for COVID-19 vaccine uptake [7]. No relevant information was found regarding consent or privacy.

#### Social and health system processes

Several studies reported that perceived secrecy and inadequate communication addressing fears and concerns about the COVID response could increase vaccine hesitancy in the population. Further, suboptimal technical/scientific communication, lack of public engagement, or lack of trust in governments and pharmaceutical companies could also contribute to vaccine hesitancy [6,7,13,14,18,21]. A study from Vietnam reported strong physician recommendations supported COVID-19 vaccine uptake amongst the population subgroups [20]. One of the studies reported that there could be unvaccinated or under-vaccinated people within larger communities of vaccinated individuals; and a greater understanding and engagement in these localised pockets would be required to allay specific fears or concerns of such groups [7].

### Strategies to improve COVID-19 vaccine acceptance and decrease hesitancy

The results indicated limited evidence on strategies to address COVID-19 vaccine hesitancy. Findings from the included studies suggest that there is no ‘one-size-fits-all’ approach to increase vaccine acceptance. We found no studies assessing strategies to address COVID vaccine acceptance or hesitancy; therefore, a summary of the recommendations from studies of determinants has been provided [7,8,13-23]. Most of the relevant literature is focussed on social and health system processes; accessibility, acceptability, affordability, and quality overall; and the vaccine experience.

Studies recommended a multi-pronged strategy to address vaccine hesitancy and promote vaccine uptake. Strategies to address vaccine hesitancy included engagement of community leaders, community mobilisation, training and education of health care professionals, nonfinancial incentives, and mass media campaigns, including efforts to increase knowledge and awareness about vaccines and vaccination [15].

#### Supply side dimensions of vaccination experience (consent, privacy, doctor-patient relationship)

Clear, consistent, and transparent communication on how vaccines are developed, how they work, their effectiveness and safety is associated with greater confidence in COVID-19 vaccines, particularly among the general population and efforts towards these may increase vaccine acceptance [6,14-16,22].

Evidence suggests that direct engagement of community mobilisers and frontline workers with the community through consultations, faith leaders and religious meetings, in simple non-medical terms helped increase vaccine acceptance [6,16,21,22,24]. Financial and non-financial incentives to address vaccine-associated costs such as travel costs or workday loss increased the chance of vaccine acceptance [17] The transportation and COVID-19 vaccination centres should be secured with adequate infection control measures to address any fears related to COVID infection spread [13]. To increase available human resources, an increase in vaccine delivery capacity through online registries is required [16]. Finally, sharing personal knowledge about being immunised and immunising by health care providers with their family members or relatives led to encouraging vaccine uptake [20].

#### Social and health system processes

Trust in governments may increase vaccine acceptance and contribute to public compliance with vaccination [6,8]. Addressing historic issues breeding distrust and being sensitive to religious and philosophical beliefs was also considered an important strategy in increasing vaccine acceptance [7]. Influential opinion leaders, including celebrities and social media influencers, were recommended to promote COVID-19 vaccination acceptance and uptake. Health and scientific experts should be used to communicate appropriate information on the safety and efficacy of vaccines [16,22,24]. Intensive campaigns to address the risk perception of COVID-19 infection and strategies that convey the emotional and immediate economic benefits of the COVID-19 vaccine were recommended to promote vaccine acceptance [21]. Vaccine communication strategies that consider the level of health, scientific and general literacy [6], in diverse populations (ie, younger, female, ethnically or linguistically diverse) [8] were found to increase vaccine acceptance. The use of traditional media (eg, television, radio, newspapers, etc) and social media platforms could be used to raise public awareness of the benefits of the COVID-19 vaccine [16,21].

### A snapshot of the data from preprint articles relevant to LMIC context

Recent surveys on vaccine acceptance and hesitancy relevant to the LMIC context were searched, and 10 surveys conducted between June 2020 and March 2021 that were due for peer review in the form of pre-prints were identified and reviewed [25-34]. The findings from these surveys were summarized to provide a snapshot of the emerging evidence, with the caveat that the analysis may change, as the papers may be revised in the future.

The findings from the surveys emphasise the need for tailored strategies to address determinants of vaccine hesitancy in several LMICs. Two surveys from India [27,29] and one from Pakistan [30] showed lower rates of vaccine hesitancy, both in the general population (17%) and in health care workers (10.6% and 0.05%, respectively), compared to other LMICs such as Bangladesh. Two surveys from Bangladesh reported the prevalence of vaccine hesitancy ranging from 32.5% to 41% [25,28]. A multinational survey conducted in LMICs reported average acceptance across studies as 80.3% [26]. The average acceptability in India was 84.6%, with 80.8% prevalence reported in females and 85.6% in males [26]. Another multinational survey reported that willingness to get vaccinated declined among respondents in LMIC such as Pakistan, from Jul 2020 through to March 2021 [33]. However, in other LMIC such as India and Vietnam, the proportion of those intended to vaccinate remained relatively stable among respondents [33].

Overall, recent findings suggested that hesitancy was high among respondents who were females, those with lower educational level, and those who were unemployed or from low-income families [25-34]. Negative attitudes towards vaccine and conspiracy beliefs towards the COVID-19 vaccine related to the perceived severity of the COVID-19 and perceived benefits of COVID-19 vaccination increased hesitancy. Increasing knowledge and awareness about the vaccine and the vaccination process was found to decrease vaccine hesitancy [26,28,33,34] Health care workers across all regions and contexts were the most trusted source of information about vaccines and their efficacy [26]. The surveys recommended clear communication by the government, using the experience of health care workers as trusted sources of medical information to address vaccine hesitancy [25,26,28,33,34].

## DISCUSSION

The review provides an insight into the level of COVID-19 vaccine acceptance and hesitancy and their determinants across LMICs. It lays out evidence from LMICs, including India, in relation to the WHO framework to understand COVID vaccine acceptance and hesitancy. The results showed that vaccine hesitancy is universal across countries and subgroups, including health care providers and the general population. Perceived disease severity, infection risk, and vaccine safety and effectiveness were some of the commonly identified determinants of vaccine acceptance. The finding of males more likely to report vaccine acceptance than females across studies may be explained by a range of social and contextual factors (eg, current or planned pregnancy) [6-8,14,17]. It will therefore be important to understand why females have lower vaccine acceptance rates. It is recommended that governments and policy makers build COVID-19 vaccination trust among the general public through timely and clear messages that advocate safety and efficacy of COVID-19 vaccines as studies have shown these to be effective [6-8,14,17,18]. Since the completion of this rapid review, another large multinational survey conducted in LMIC was published, which reported similar findings [35]. It was found that vaccine acceptance was also positively associated with COVID-19 knowledge, higher income, younger age, and testing negative for COVID-19. Female gender and chronic disease were associated with reduced odds for vaccine acceptance [35].

It is somewhat reassuring that vaccine hesitancy on the whole in LMICs remains comparatively low as compared to HICs [6]. A high COVID-19 vaccine acceptance level in the overall Indian population coupled with hesitancy in certain population subgroups [6,7,14,27,29] pointed towards need to explore the causes or contexts of this hesitancy. Studies also suggest that localised pockets of hesitancy in otherwise well-vaccinated populations may emerge as an additional challenge. The finding that trust in information and sources plays a role in increasing acceptance [7,8,15,18,20], is highly relevant and needs to be leveraged for increasing acceptance.

Policy-makers at national and subnational levels may consider the following key determinants of vaccine acceptance, which likely help in developing tailored strategies. These include risk perception and severity of illness, gender, occupation, education, income, place of residence, and religious beliefs. Vaccine-related determinants include vaccine effectiveness, side effects, and perceptions of safety including exposure risks while getting vaccinated. Other key determinants include misinformation and affordability; endorsement from health providers and employers; communication and public engagement; and a lack of trust in government sources and pharmaceutical companies.

There is a lack of sufficient evidence on strategies to increase vaccine acceptance and decrease vaccine hesitancy in the LMIC context. However, this issue has been addressed previously, as in the case of childhood and other types of immunisation in countries like India and there is requirement of similar efforts [29,30]. Trust in information and sources, engaging influential leaders, political commitment, improved communication including addressing vaccine safety concerns, tailoring immunisation programs to local contexts, partnering with other vaccine providers in the communities, education and creating locally relevant awareness initiatives play a role in increasing vaccine acceptance and decreasing hesitancy [15,36-42]. We may also consider adaptation of approaches used in other HIC settings like Israel [43]. The study recommends some key strategies to increase vaccination rates, especially in younger and healthier individuals who are more reluctant to be vaccinated. The Israel Ministry of Health addressed the issue of vaccine hesitancy in several ways, including the opening of vaccination centres at night, removing the need for pre-registration, setting up vaccine carts in nature reserves on weekends and offering free meals as incentives. It is important to inform the public of the initial results of real-life impact and effectiveness in real-time, as clear and transparent communication may increase public trust leading towards higher vaccination rates [43].

Multipronged strategies should be considered. These may include minimisation of vaccine-associated costs borne by the public; measures to ensure infection control and reduce infection spread; increase in the availability of human resources and vaccine delivery capacity; direct engagement with communities by mobilisers and frontline workers, and providers sharing knowledge and encouraging vaccination in their personal networks; and support for vaccine registration especially for those with access constraints (4,6-8,14,16,17,21,28]. Existing diagnostic and outreach facilities may be utilised for vaccine registration. Other strategies may include clear, consistent, and transparent communication regarding service availability, risks, and benefits of vaccines through general and intensive campaigns [4,6-8,14,15,17,18,20,28]. Messages should be tailored according to the health, scientific and general literacy of sub-populations using traditional and social media, as appropriate. The governments worldwide need appropriate strategies to develop plans to address the anxiety and vaccine related concerns of community leading to vaccine hesitancy [44].

Our review has some limitations. Studies retrieved from electronic databases may not provide current public opinions due to the journal publication process. However, this approach was undertaken to provide a concise summary of the evidence on COVID-19 vaccine hesitancy in the LMIC context within a short timeframe. Due to the rapid nature of the review, the inclusion of studies may not be exhaustive. The methodological quality of the included studies was not evaluated, which may have impacted the reliability of the conclusions. The included studies mainly were cross-sectional surveys that provided snapshots of vaccine hesitancy in different LMICs, which also showed that different approaches were used to evaluate the willingness to accept COVID-19 vaccines in multiple studies. Some surveys used a Likert scale and some binary response of yes/No. An in-depth comparison of differences in vaccine acceptance rates and the reasons for them between different studies was not done and can be considered as one of the limitations.

However, despite the limitations of the review, the consistency of the findings from the included studies provides an understanding of COVID-19 vaccine acceptance and hesitancy across different regions in LMIC. There is a lack of evidence on formal evaluation of strategies to address vaccine acceptance and hesitancy. There is a need to test strategies using pre-and post-test studies to address vaccine hesitancy and increase vaccine uptake in LMICs.

## CONCLUSIONS

This rapid review contributes to the evidence base for vaccine acceptance and hesitancy, with some policy implications for the LMIC context. Decision-makers should consider determinants for reluctance in the relevant context and tailor appropriate and feasible solutions to the target population. Studies suggest high acceptance for the most part; localised strategies to address concerns and misinformation are required to engage the community and are based on broader trust-building and vaccine delivery system-strengthening activities. Further inquiry into best practices for this and adaptation of known strategies and approaches from different LMICs are recommended.

## Additional material


Online Supplementary Document

## References

[1] World Health Organization. WHO Coronavirus (COVID-19) Dashboard 2021. Available: https://covid19.who.int/. Accessed: 26 May 2021.

[2] World Health Organization. COVID 19 Vaccines 2021. Available: https://www.who.int/emergencies/diseases/novel-coronavirus-2019/covid-19-vaccines. Accessed: 26 May 2021.

[3] Conte C Sogni F Affanni P Veronesi L Argentiero A Esposito S Vaccines against Coronaviruses: The State of the Art. Vaccines (Basel). 2020;8:309. 10.3390/vaccines8020309 32560340 PMC7350246

[4] Harrison EA Wu JW Vaccine confidence in the time of COVID-19. Eur J Epidemiol. 2020;35:325-30. 10.1007/s10654-020-00634-3 32318915 PMC7174145

[5] Bartsch SM O’Shea KJ Ferguson MC Bottazzi ME Wedlock PT Strych U Vaccine Efficacy Needed for a COVID-19 Coronavirus Vaccine to Prevent or Stop an Epidemic as the Sole Intervention. Am J Prev Med. 2020;59:493-503. 10.1016/j.amepre.2020.06.011 32778354 PMC7361120

[6] Lazarus JV Ratzan SC Palayew A Gostin LO Larson HJ Rabin K A global survey of potential acceptance of a COVID-19 vaccine. Nat Med. 2021;27:225-8. 10.1038/s41591-020-1124-9 33082575 PMC7573523

[7] Lazarus JV Wyka K Rauh L Rabin K Ratzan S Gostin LO Hesitant or Not? The Association of Age, Gender, and Education with Potential Acceptance of a COVID-19 Vaccine: A Country-level Analysis. J Health Commun. 2020;25:799-807. 10.1080/10810730.2020.1868630 33719881

[8] Lin C Tu P Beitsch LM Confidence and Receptivity for COVID-19 Vaccines: A Rapid Systematic Review. Vaccines (Basel). 2020;9:16. 10.3390/vaccines9010016 33396832 PMC7823859

[9] Roozenbeek J Schneider CR Dryhurst S Kerr J Freeman ALJ Recchia G Susceptibility to misinformation about COVID-19 around the world. R Soc Open Sci. 2020;7:201199. 10.1098/rsos.201199 33204475 PMC7657933

[10] Our World in Data. Coronavirus (COVID-19) Vaccinations 2021. Available: https://ourworldindata.org/covid-vaccinations?country=OWID_WRL. Accessed: 14 June 2021.

[11] The New York Times. Tracking Coronavirus Vaccinations Around the World 2021 Available: https://www.nytimes.com/interactive/2021/world/covid-vaccinations-tracker.html. Accessed: 14 June 2021.

[12] Government of India. National Co-Win Statistics 2021 [cited 2021 28 May]. Available: https://dashboard.cowin.gov.in/.

[13] Bhopal S Nielsen M Vaccine hesitancy in low- and middle-income countries: potential implications for the COVID-19 response. Arch Dis Child. 2021;106:113-4. 10.1136/archdischild-2020-318988 32912868

[14] Sallam M Covid-19 vaccine hesitancy worldwide: A concise systematic review of vaccine acceptance rates. Vaccines (Basel). 2021;9:1-15. 33669441 10.3390/vaccines9020160 PMC7920465

[15] World Health Organization. Improving vaccination demand and addressing hesitancy 2020. Available: https://www.who.int/teams/immunization-vaccines-and-biologicals/essential-programme-on-immunization/demand. Accessed: 29 April 2021.

[16] Hasan T Beardsley J Marais BJ Nguyen TA Fox GJ The implementation of mass-vaccination against SARS-CoV-2: A systematic review of existing strategies and guidelines. Vaccines (Basel). 2021;9:326. 10.3390/vaccines9040326 33915829 PMC8066252

[17] Abedin M Islam MA Rahman FN Reza HM Hossain MZ Hossain MA Willingness to vaccinate against COVID-19 among Bangladeshi adults: Understanding the strategies to optimize vaccination coverage. PLoS One. 2021;16:e0250495. 10.1371/journal.pone.0250495 33905442 PMC8078802

[18] Adebisi YA Alaran AJ Bolarinwa OA Akande-Sholabi B Lucero-Prisno DE When it is available, will we take it? Social media users’ perception of hypothetical covid-19 vaccine in nigeria. Pan Afr Med J. 2021;38:230. . 10.11604/pamj.2021.38.230.27325 34046135 PMC8140724

[19] Al-Qerem WA Jarab AS COVID-19 Vaccination Acceptance and Its Associated Factors Among a Middle Eastern Population. Front Public Health. 2021;9:632914. 10.3389/fpubh.2021.632914 33643995 PMC7902782

[20] Huynh G Tran TT Nguyen HTN Pham LA COVID-19 vaccination intention among healthcare workers in Vietnam. Asian Pac J Trop Med. 2021;14:159-64. 10.4103/1995-7645.312513

[21] Dzinamarira T Nachipo B Phiri B Musuka G Covid-19 vaccine roll-out in south africa and zimbabwe: Urgent need to address community preparedness, fears and hesitancy. Vaccines (Basel). 2021;9:1-10. 33809002 10.3390/vaccines9030250 PMC8000117

[22] Ministry of Health and Family Welfare. COVID-19 Vaccine Communication Strategy. 2020.

[23] Ministry of Health and Family Welfare. COVID-19 Vaccines Operational Guidelines 2020. Available: https://www.mohfw.gov.in/covid_vaccination/vaccination/important-information.html. Accessed: 4 May 2021.

[24] Khan YH Mallhi TH Alotaibi NH Alzarea AI Alanazi AS Tanveer N Threat of COVID-19 Vaccine Hesitancy in Pakistan: The Need for Measures to Neutralize Misleading Narratives. Am J Trop Med Hyg. 2020;103:603-4. 10.4269/ajtmh.20-0654 32588810 PMC7410483

[25] Ali M Hossain A What is the extent of COVID-19 vaccine hesitancy in Bangladesh?: A cross-sectional rapid national survey. medRxiv. 2021:2021.02.17.21251917. 10.1136/bmjopen-2021-050303 PMC8387740 34429316

[26] Arce JSS Warren SS Meriggi NF Scacco A McMurry N Voors M COVID-19 Vaccine Acceptance and Hesitancy in Low and Middle Income Countries, and Implications for Messaging. medRxiv. 2021:2021.03.11.21253419.

[27] Gautam A Dhara B Mukherjee D Mukhopadhyay D Roy S Ganguly SS A Digital Survey on the Acceptance and Affordability of COVID 19 Vaccine among the People of West Bengal, India- A Survey Based Study. medRxiv. 2020:2020.11.13.20229534.

[28] Hossain MB Alam MZ Islam MS Sultan S Faysal MM Rima S COVID-19 Vaccine Hesitancy among the Adult Population in Bangladesh: A Nationally Representative Cross-sectional Survey. medRxiv. 2021:2021.04.23.21255844. 10.1371/journal.pone.0260821 PMC8659424 34882726

[29] Jain J Saurabh S Goel AD Gupta MK Bhardwaj P Raghav PR COVID-19 vaccine hesitancy among undergraduate medical students: results from a nationwide survey in India. medRxiv. 2021:2021.03.12.21253444.

[30] Malik A Malik J Ishaq U Acceptance of COVID-19 Vaccine in Pakistan Among Health Care Workers. medRxiv. 2021:2021.02.23.21252271. 10.1371/journal.pone.0257237 PMC8443053 34525110

[31] Dereje N Tesfaye A Tamene B Alemeshet D Abe H Tesfa N COVID-19 Vaccine hesitancy in Addis Ababa, Ethiopia: A mixed-methods study. medRxiv. 2021:2021.02.25.21252443. 10.1136/bmjopen-2021-052432 PMC9152622 35636790

[32] Islam F Agarwalla R Panda M Alvi Y Singh V Debroy A Assessment of the knowledge, preferences and concern regarding the prospective COVID-19 vaccine among adults residing in New Delhi, India-A cross sectional study. medRxiv. 2021:2021.01.23.21250164. 10.4103/jfmpc.jfmpc_2437_20 PMC8284199 34322440

[33] Kothari A Pfuhl G Schieferdecker D Harris CT Tidwell C Fitzpatrick KM The Barrier to Vaccination Is Not Vaccine Hesitancy: Patterns of COVID-19 Vaccine Acceptance over the Course of the Pandemic in 23 Countries. medRxiv. 2021:2021.04.23.21253857.

[34] Olomofe CO Soyemi VK Udomah BF Owolabi AO Ajumuka EE Igbokwe CM Predictors of uptake of a potential covid-19 vaccine among nigerian adults. medRxiv. 2021:2020.12.28.20248965.

[35] Bono SA Faria de Moura Villela E Siau CS Chen WS Pengpid S Hasan MT Factors Affecting COVID-19 Vaccine Acceptance: An International Survey among Low- and Middle-Income Countries. Vaccines (Basel). 2021;9:515. 10.3390/vaccines9050515 34067682 PMC8157062

[36] Agrawal A Kolhapure S Di Pasquale A Rai J Mathur A Vaccine Hesitancy as a Challenge or Vaccine Confidence as an Opportunity for Childhood Immunisation in India. Infect Dis Ther. 2020;9:421-32. 10.1007/s40121-020-00302-9 32447713 PMC7452967

[37] Jalloh MF Jalloh MB Albert A Wolff B Callis A Ramakrishnan A Perceptions and acceptability of an experimental Ebola vaccine among health care workers, frontline staff, and the general public during the 2014-2015 Ebola outbreak in Sierra Leone. Vaccine. 2019;37:1495-502. 10.1016/j.vaccine.2019.01.046 30755367 PMC7393388

[38] Kpanake L Sorum PC Mullet É Willingness to get vaccinated against Ebola: A mapping of Guinean people positions. Hum Vaccin Immunother. 2018;14:2391-6. 10.1080/21645515.2018.1480236 29923787 PMC6284512

[39] Nguyen T Henningsen KH Brehaut JC Hoe E Wilson K Acceptance of a pandemic influenza vaccine: a systematic review of surveys of the general public. Infect Drug Resist. 2011;4:197-207. 22114512 10.2147/IDR.S23174 PMC3215344

[40] Sankaranarayanan R Basu P Kaur P Bhaskar R Singh GB Denzongpa P Current status of human papillomavirus vaccination in India’s cervical cancer prevention efforts. Lancet Oncol. 2019;20:e637-44. 10.1016/S1470-2045(19)30531-5 31674322

[41] Butler R MacDonald NE Diagnosing the determinants of vaccine hesitancy in specific subgroups: The Guide to Tailoring Immunization Programmes (TIP). Vaccine. 2015;33:4176-9. 10.1016/j.vaccine.2015.04.038 25896376

[42] Woods LO Bridges CB Graitcer SB Lamont BUS Immunization program adult immunization activities and resources. Hum Vaccin Immunother. 2016;12:1045-50. 10.1080/21645515.2015.1109756 26577532 PMC4962972

[43] Shilo S Rossman H Segal E Signals of hope: gauging the impact of a rapid national vaccination campaign. Nat Rev Immunol. 2021;21:198-9. 10.1038/s41577-021-00531-0 33712744 PMC7953505

[44] Yadav A Ghosh S Kotwal A COVID-19 vaccines- panacea or delusion: A public health perspective. Journal of Marine Medical Society. 2020;22:110-2. 10.4103/jmms.jmms_181_20

