# Supplementary material for: A rapid review of evidence on the determinants of and strategies for COVID-19 vaccine acceptance in low- and middle-income countries
Source: J Glob Health. 2021 Nov 20;11:05027. doi: 10.7189/jogh.11.05027 (PMC8645216; doi:10.7189/jogh.11.05027)
Supplement: Online Supplementary Document [file jogh-11-05027-s001.pdf]

Appendices for Determinants of and Strategies for COVID-19 Vaccine Acceptance: A Rapid Evidence Synthesis

Appendix S1 – Search Strategies

PubMed

No.	Search terms	Number of hits
#1	“vaccines”[MH] OR “vaccin”[tw] OR “vaccination”[MH] OR “vaccination”[tw] “vaccinate”[tw] OR “vaccinated”[tw] OR “vaccinations”[tw] OR “vaccines”[tw] OR “vaccine”[tw] OR immunis*[tw] OR immuniz*[tw]	446,542
#2	(“2019-nCoV” OR “2019nCoV” OR “COVID-19” OR “SARS-CoV-2” OR “SARS-2”) OR (wuhan AND coronavirus) OR ("COVID-19"[Supplementary Concept] OR "severe acute respiratory syndrome coronavirus 2"[Supplementary Concept]) OR “Wuhan seafood market pneumonia virus”	125,053
#3	Hemorrhagic Fever, Ebola[MH] OR Ebola[tw] OR SARS[tw] OR coronavirus[tw] OR “severe acute respiratory syndrome” [tw] OR “SARS Virus”[MH] OR “SARS Virus”[tw] OR “SARS-CoV”[tw] OR “SARS-Associated Coronavirus”[tw] OR “sudden acute respiratory syndrome”[tw] OR “swine flu”[tw] OR “swine influenza”[tw] OR “HIN1”[tw] OR pandemic[tw] OR pandemics[tw] OR “disease outbreak”[tw] OR “disease outbreaks”[tw] OR outbreak[tw] OR “outbreaks”[tw]	238,800
#4	#2 OR #3	302,494
#5	Accept*[tw] OR confidence[tw] OR uptake[tw] OR participat*[tw] OR improv*[tw] OR hesitanc*[tw]	4,368,824
#6	afghanistan[MeSH] OR albania[MeSH] OR algeria[MeSH] OR american samoa[MeSH] OR angola[MeSH] OR antigua and barbuda[MeSH] OR argentina[MeSH] OR armenia[MeSH] OR aruba[MeSH] OR azerbaijan[MeSH] OR bahrain[MeSH] OR bangladesh[MeSH] OR barbados[MeSH] OR republic of belarus[MeSH] OR belize[MeSH] OR benin[MeSH] OR bhutan[MeSH] OR bolivia[MeSH] OR bosnia and herzegovina[MeSH] OR botswana[MeSH] OR brazil[MeSH] OR bulgaria[MeSH] OR burkina faso[MeSH] OR burundi[MeSH] OR cabo verde[MeSH] OR cambodia[MeSH] OR cameroon[MeSH] OR central african republic[MeSH] OR chad[MeSH] OR chile[MeSH] OR china[MeSH] OR colombia[MeSH] OR comoros[MeSH] OR democratic republic of the congo[MeSH] OR congo[MeSH] OR costa rica[MeSH] OR cote d’ivoire[MeSH] OR croatia[MeSH] OR cuba[MeSH] OR cyprus[MeSH] OR czech republic[MeSH] OR djibouti[MeSH] OR dominica[MeSH] OR dominican republic[MeSH] OR ecuador[MeSH] OR egypt[MeSH] OR el salvador[MeSH] OR equatorial guinea[MeSH] OR eritrea[MeSH] OR estonia[MeSH] OR swaziland[MeSH] OR ethiopia[MeSH] OR fiji[MeSH] OR gabon[MeSH] OR gambia[MeSH] OR georgia (republic)[MeSH] OR ghana[MeSH] OR gibraltar[MeSH] OR greece[MeSH] OR grenada[MeSH] OR guam[MeSH] OR guatemala[MeSH] OR guinea[MeSH] OR guinea bissau[MeSH] OR guyana[MeSH] OR haiti[MeSH] OR honduras[MeSH] OR hungary[MeSH] OR	1,411,106

	india[MeSH] OR indonesia[MeSH] OR iran[MeSH] OR iraq[MeSH] OR jamaica[MeSH] OR jordan[MeSH] OR kazakhstan[MeSH] OR kenya[MeSH] OR democratic people's republic of korea[MeSH] OR republic of korea[MeSH] OR kosovo[MeSH] OR kyrgyzstan[MeSH] OR laos[MeSH] OR latvia[MeSH] OR lebanon[MeSH] OR lesotho[MeSH] OR liberia[MeSH] OR libya[MeSH] OR lithuania[MeSH] OR macau[MeSH] OR republic of north macedonia[MeSH] OR madagascar[MeSH] OR malawi[MeSH] OR malaysia[MeSH] OR indian ocean islands[MeSH] OR mali[MeSH] OR malta[MeSH] OR micronesia[MeSH] OR palau[MeSH] OR mauritania[MeSH] OR mauritius[MeSH] OR mexico[MeSH] OR moldova[MeSH] OR mongolia[MeSH] OR montenegro[MeSH] OR morocco[MeSH] OR mozambique[MeSH] OR myanmar[MeSH] OR namibia[MeSH] OR nepal[MeSH] OR netherlands antilles[MeSH] OR nicaragua[MeSH] OR niger[MeSH] OR nigeria[MeSH] OR oman[MeSH] OR pakistan[MeSH] OR panama[MeSH] OR papua new guinea[MeSH] OR paraguay[MeSH] OR peru[MeSH] OR philippines[MeSH] OR poland[MeSH] OR portugal[MeSH] OR puerto rico[MeSH] OR romania[MeSH] OR russia[MeSH] OR rwanda[MeSH] OR samoa[MeSH] OR sao tome and principe[MeSH] OR saudi arabia[MeSH] OR senegal[MeSH] OR serbia[MeSH] OR seychelles[MeSH] OR sierra leone[MeSH] OR slovakia[MeSH] OR slovenia[MeSH] OR melanesia[MeSH] OR somalia[MeSH] OR south africa[MeSH] OR south sudan[MeSH] OR sri lanka[MeSH] OR saint kitts and nevis[MeSH] OR saint lucia[MeSH] OR saint vincent and the grenadines[MeSH] OR sudan[MeSH] OR suriname[MeSH] OR syria[MeSH] OR tajikistan[MeSH] OR tanzania[MeSH] OR thailand[MeSH] OR timor leste[MeSH] OR togo[MeSH] OR tonga[MeSH] OR trinidad and tobago[MeSH] OR tunisia[MeSH] OR turkey[MeSH] OR turkmenistan[MeSH] OR uganda[MeSH] OR ukraine[MeSH] OR uruguay[MeSH] OR uzbekistan[MeSH] OR vanuatu[MeSH] OR venezuela[MeSH] OR vietnam[MeSH] OR middle east[MeSH] OR yemen[MeSH] OR yugoslavia[MeSH] OR zambia[MeSH] OR zimbabwe[MeSH] OR africa south of the sahara[MeSH] OR africa, central[MeSH] OR africa, northern[MeSH] OR africa, southern[MeSH] OR africa, eastern[MeSH] OR africa, western[MeSH] OR west indies[MeSH] OR indian ocean islands[MeSH] OR caribbean region[MeSH] OR central america[MeSH] OR latin america[MeSH] OR south america[MeSH] OR asia, central[MeSH] OR asia, northern[MeSH] OR asia, southeastern[MeSH] OR asia, western[MeSH] OR europe, eastern[MeSH] OR developing countries[MeSH]	
#7	afghanistan[tiab] OR albania[tiab] OR algeria[tiab] OR american samoa[tiab] OR angola[tiab] OR antigua[tiab] OR barbuda[tiab] OR argentina[tiab] OR armenia[tiab] OR armenian[tiab] OR aruba[tiab] OR azerbaijan[tiab] OR bahrain[tiab] OR bangladesh[tiab] OR barbados[tiab] OR belarus[tiab] OR byelarus[tiab] OR belorussia[tiab] OR byelorussian[tiab] OR belize[tiab] OR british honduras[tiab] OR benin[tiab] OR dahomey[tiab] OR bhutan[tiab] OR bolivia[tiab] OR bosnia[tiab] OR herzegovina[tiab] OR botswana[tiab] OR bechuanaland[tiab] OR brazil[tiab] OR brasil[tiab] OR bulgaria[tiab] OR burkina faso[tiab] OR burkina fasso[tiab] OR upper volta[tiab] OR burundi[tiab] OR urundi[tiab] OR cabo verde[tiab] OR cape verde[tiab] OR cambodia[tiab] OR kampuchea[tiab] OR khmer republic[tiab] OR cameroon[tiab] OR cameron[tiab] OR cameroun[tiab] OR central african republic[tiab] OR ubangi shari[tiab] OR chad[tiab] OR chile[tiab] OR china[tiab] OR colombia[tiab] OR comoros[tiab] OR comoro islands[tiab] OR	1,490,341

	mayotte[tiab] OR congo[tiab] OR zaire[tiab] OR costa rica[tiab] OR cote d'ivoire[tiab] OR cote d' ivoire[tiab] OR cote divoire[tiab] OR cote d ivoire[tiab] OR ivory coast[tiab] OR croatia[tiab] OR cuba[tiab] OR cyprus[tiab] OR czech republic[tiab] OR czechoslovakia[tiab] OR djibouti[tiab] OR french somaliland[tiab] OR dominica[tiab] OR dominican republic[tiab] OR ecuador[tiab] OR egypt[tiab] OR united arab republic[tiab] OR el salvador[tiab] OR equatorial guinea[tiab] OR spanish guinea[tiab] OR eritrea[tiab] OR estonia[tiab] OR eswatini[tiab] OR swaziland[tiab] OR ethiopia[tiab] OR fiji[tiab] OR gabon[tiab] OR gabonese republic[tiab] OR gambia[tiab] OR georgia[tiab] OR georgian[tiab] OR ghana[tiab] OR gold coast[tiab] OR gibraltar[tiab] OR greece[tiab] OR grenada[tiab] OR guam[tiab] OR guatemala[tiab] OR guinea[tiab] OR guyana[tiab] OR guiana[tiab] OR haiti[tiab] OR hispaniola[tiab] OR honduras[tiab] OR hungary[tiab] OR india[tiab] OR indonesia[tiab] OR timor[tiab] OR iran[tiab] OR iraq[tiab] OR isle of man[tiab] OR jamaica[tiab] OR jordan[tiab] OR kazakhstan[tiab] OR kazakh[tiab] OR kenya[tiab] OR korea[tiab] OR kosovo[tiab] OR kyrgyzstan[tiab] OR kirghizia[tiab] OR kirgizstan[tiab] OR kyrgyz republic[tiab] OR kirghiz[tiab] OR laos[tiab] OR lao pdr[tiab] OR lao people's democratic republic[tiab] OR latvia[tiab] OR lebanon[tiab] OR lesotho[tiab] OR basutoland[tiab] OR liberia[tiab] OR libya[tiab] OR libyan arab jamahiriya[tiab] OR lithuania[tiab] OR macau[tiab] OR macao[tiab] OR macedonia[tiab] OR madagascar[tiab] OR malagasy republic[tiab] OR malawi[tiab] OR nyasaland[tiab] OR malaysia[tiab] OR maldives[tiab] OR indian ocean[tiab] OR mali[tiab] OR malta[tiab] OR micronesia[tiab] OR kiribati[tiab] OR marshall islands[tiab] OR nauru[tiab] OR northern mariana islands[tiab] OR palau[tiab] OR tuvalu[tiab] OR mauritania[tiab] OR mauritius[tiab] OR mexico[tiab] OR moldova[tiab] OR moldovian[tiab] OR mongolia[tiab] OR montenegro[tiab] OR morocco[tiab] OR ifni[tiab] OR mozambique[tiab] OR portuguese east africa[tiab] OR myanmar[tiab] OR burma[tiab] OR namibia[tiab] OR nepal[tiab] OR netherlands antilles[tiab] OR nicaragua[tiab] OR niger[tiab] OR nigeria[tiab] OR oman[tiab] OR muscat[tiab] OR pakistan[tiab] OR panama[tiab] OR papua new guinea[tiab] OR paraguay[tiab] OR peru[tiab] OR philippines[tiab] OR philipines[tiab] OR phillipines[tiab] OR phillippines[tiab] OR poland[tiab] OR polish people's republic[tiab] OR portugal[tiab] OR portuguese republic[tiab] OR puerto rico[tiab] OR romania[tiab] OR russia[tiab] OR russian federation[tiab] OR ussr[tiab] OR soviet union[tiab] OR union of soviet socialist republics[tiab] OR rwanda[tiab] OR ruanda[tiab] OR samoa[tiab] OR pacific islands[tiab] OR polynesia[tiab] OR samoan islands[tiab] OR sao tome and principe[tiab] OR saudi arabia[tiab] OR senegal[tiab] OR serbia[tiab] OR seychelles[tiab] OR sierra leone[tiab] OR slovakia[tiab] OR slovak republic[tiab] OR slovenia[tiab] OR melanesia[tiab] OR solomon island[tiab] OR solomon islands[tiab] OR norfolk island[tiab] OR somalia[tiab] OR south africa[tiab] OR south sudan[tiab] OR sri lanka[tiab] OR ceylon[tiab] OR saint kitts and nevis[tiab] OR st kitts and nevis[tiab] OR saint lucia[tiab] OR st lucia[tiab] OR saint vincent[tiab] OR st vincent[tiab] OR grenadines[tiab] OR sudan[tiab] OR suriname[tiab] OR surinam[tiab] OR syria[tiab] OR syrian arab republic[tiab] OR tajikistan[tiab] OR tadjikistan[tiab] OR tadzhikistan[tiab] OR tadzhik[tiab] OR tanzania[tiab] OR tanganyika[tiab] OR thailand[tiab] OR siam[tiab] OR timor leste[tiab] OR east timor[tiab] OR togo[tiab] OR togolese republic[tiab] OR tonga[tiab] OR trinidad[tiab] OR tobago[tiab] OR tunisia[tiab] OR	

	turkey[tiab] OR turkmenistan[tiab] OR turkmen[tiab] OR uganda[tiab] OR ukraine[tiab] OR uruguay[tiab] OR uzbekistan[tiab] OR uzbek[tiab] OR vanuatu[tiab] OR new hebrides[tiab] OR venezuela[tiab] OR vietnam[tiab] OR viet nam[tiab] OR middle east[tiab] OR west bank[tiab] OR gaza[tiab] OR palestine[tiab] OR yemen[tiab] OR yugoslavia[tiab] OR zambia[tiab] OR zimbabwe[tiab] OR northern rhodesia[tiab] OR global south[tiab] OR africa south of the sahara[tiab] OR sub saharan africa[tiab] OR subsaharan africa[tiab] OR central africa[tiab] OR north africa[tiab] OR northern africa[tiab] OR magreb[tiab] OR maghrib[tiab] OR sahara[tiab] OR southern africa[tiab] OR east africa[tiab] OR eastern africa[tiab] OR west africa[tiab] OR western africa[tiab] OR west indies[tiab] OR indian ocean islands[tiab] OR caribbean[tiab] OR central america[tiab] OR latin america[tiab] OR south america[tiab] OR central asia[tiab] OR north asia[tiab] OR northern asia[tiab] OR southeastern asia[tiab] OR south eastern asia[tiab] OR southeast asia[tiab] OR south east asia[tiab] OR western asia[tiab] OR east europe[tiab] OR eastern europe[tiab] OR developing country[tiab] OR developing countries[tiab] OR developing nation[tiab] OR developing nations[tiab] OR developing population[tiab] OR developing populations[tiab] OR developing world[tiab] OR less developed country[tiab] OR less developed countries[tiab] OR less developed nation[tiab] OR less developed nations[tiab] OR less developed world[tiab] OR lesser developed countries[tiab] OR lesser developed nations[tiab] OR under developed country[tiab] OR under developed countries[tiab] OR under developed nations[tiab] OR under developed world[tiab] OR underdeveloped country[tiab] OR underdeveloped countries[tiab] OR underdeveloped nation[tiab] OR underdeveloped nations[tiab] OR underdeveloped population[tiab] OR underdeveloped populations[tiab] OR underdeveloped world[tiab] OR middle income country[tiab] OR middle income countries[tiab] OR middle income nation[tiab] OR middle income nations[tiab] OR middle income population[tiab] OR middle income populations[tiab] OR low income country[tiab] OR low income countries[tiab] OR low income nation[tiab] OR low income nations[tiab] OR low income population[tiab] OR low income populations[tiab] OR lower income country[tiab] OR lower income countries[tiab] OR lower income nations[tiab] OR lower income population[tiab] OR lower income populations[tiab] OR underserved countries[tiab] OR underserved nations[tiab] OR underserved population[tiab] OR underserved populations[tiab] OR under served population[tiab] OR under served populations[tiab] OR deprived countries[tiab] OR deprived population[tiab] OR deprived populations[tiab] OR poor country[tiab] OR poor countries[tiab] OR poor nation[tiab] OR poor nations[tiab] OR poor population[tiab] OR poor populations[tiab] OR poor world[tiab] OR poorer countries[tiab] OR poorer nations[tiab] OR poorer population[tiab] OR poorer populations[tiab] OR developing economy[tiab] OR developing economies[tiab] OR less developed economy[tiab] OR less developed economies[tiab] OR underdeveloped economies[tiab] OR middle income economy[tiab] OR middle income economies[tiab] OR low income economy[tiab] OR low income economies[tiab] OR lower income economies[tiab] OR low gdp[tiab] OR low gnp[tiab] OR low gross domestic[tiab] OR low gross national[tiab] OR lower gdp[tiab] OR lower gross domestic[tiab] OR lmic[tiab] OR lmics[tiab] OR third world[tiab] OR lami country[tiab] OR lami countries[tiab] OR	

	transitional country[tiab] OR transitional countries[tiab] OR emerging economies[tiab] OR emerging nation[tiab] OR emerging nations[tiab]	
#8	#6 OR #7	1,991,740
#9	#1 AND #4 AND #5 AND #8	2,142
#10	#1 AND #2 Filters: Humans; English	1,443

EMBASE

No.	Search terms	Number of hits
#1	vaccine OR vaccine/exp OR vaccination OR vaccination/exp OR immunization OR immunization/exp OR immunisation	610,644
#2	((“2019-nCoV” OR “2019nCoV” OR COVID-19/exp OR "COVID-19" OR “SARS-CoV-2” OR “SARS-2” O "severe acute respiratory syndrome coronavirus 2" OR (wuhan AND coronavirus))) OR “Wuhan seafood market pneumonia virus”	10,981
#3	Ebola hemorrhagic fever/de OR Ebola OR SARS OR coronavirus OR “severe acute respiratory syndrome”/de OR “severe acute respiratory syndrome” OR “SARS Virus” OR “SARS Virus” OR “SARS-CoV” OR “SARS-Associated Coronavirus” OR “sudden acute respiratory syndrome” OR “swine flu” OR “swine influenza”/de OR “swine influenza” OR “HIN1” OR pandemic/de OR pandemic* OR “disease outbreak” OR “disease outbreaks” OR outbreak OR “outbreaks”	298,307
#4	#2 OR #3	298,307
#5	(acceptance OR accept OR uptake OR confidence OR participat* OR improve OR hesitancy OR hesitant OR intent OR intention):ti,ab,kw	3,250,151
#6	(Afghanistan OR Albania OR Algeria OR american samoa OR angola OR "antigua and barbuda" OR argentina OR Armenia OR aruba OR azerbaijan OR Bahrain OR Bangladesh OR Barbados OR Belarus OR belize OR benin OR bhutan OR bolivia OR "bosnia and herzegovina" OR botswana OR brazil OR bulgaria OR burkina faso OR burundi OR cape verde OR cambodia OR cameroon OR central african republic OR chad OR chile OR china OR colombia OR comoros OR democratic republic congo OR congo OR costa rica OR "cote d ivoire" OR croatia OR cuba OR cyprus OR czech republic OR djibouti OR dominica OR dominican republic OR ecuador OR egypt OR el salvador OR equatorial guinea OR eritrea OR estonia OR swaziland OR ethiopia OR fiji OR gabon OR gambia OR "georgia (republic)" OR ghana OR gibraltar OR greece OR grenada OR guam OR guatemala OR guinea OR guinea bissau OR guyana OR haiti OR honduras OR hungary OR india OR indonesia OR iran OR iraq OR isle of man OR jamaica OR jordan OR kazakhstan OR kenya OR north korea OR south korea OR korea OR kosovo OR kyrgyzstan OR laos OR latvia OR lebanon OR lesotho OR liberia OR libyan arab jamahiriya OR lithuania OR macau OR republic of north macedonia OR madagascar OR malawi OR malaysia OR indian ocean OR mali OR malta OR federated states of micronesia OR kiribati OR mauritania OR mauritius OR mexico OR moldova OR mongolia OR "montenegro (republic)" OR morocco OR mozambique OR myanmar OR namibia OR nepal OR netherlands antilles OR nicaragua OR niger OR nigeria OR oman OR pakistan OR panama OR papua new guinea OR paraguay OR peru OR philippines OR poland OR portugal OR puerto rico OR romania OR russian federation OR rwanda OR samoa OR "sao tome and principe" OR saudi arabia OR senegal OR serbia OR	98,978

	seychelles OR sierra leone OR slovakia OR slovenia OR melanesia OR somalia OR south africa OR south sudan OR sri lanka OR "saint kitts and nevis" OR saint lucia OR "saint vincent and the grenadines" OR sudan OR suriname OR syrian arab republic OR tajikistan OR tanzania OR thailand OR timor leste OR togo OR tonga OR "trinidad and tobago" OR tunisia OR "turkey republic" OR turkmenistan OR uganda OR ukraine OR uruguay OR uzbekistan OR vanuatu OR venezuela OR viet nam OR palestine OR yemen OR yugoslavia OR zambia OR zimbabwe OR africa south of the sahara OR africa, central OR africa, northern OR africa, southern OR africa, eastern OR africa, western OR west indies OR indian ocean islands OR caribbean region OR central america OR south america OR asia, central OR asia, northern OR asia, southeastern OR asia, western OR europe, eastern OR developing country):de	
#7	(afghanistan OR albania OR algeria OR "american samoa" OR angola OR "antigua and barbuda" OR antigua OR barbuda OR argentina OR armenia OR armenian OR aruba OR azerbaijan OR bahrain OR bangladesh OR barbados OR republic of belarus OR belarus OR byelarus OR belorussia OR byelorussian OR belize OR "british honduras" OR benin OR dahomey OR bhutan OR bolivia OR "bosnia and herzegovina" OR bosnia OR herzegovina OR botswana OR bechuanaland OR brazil OR brasil OR bulgaria OR "burkina faso" OR "burkina fasso" OR "upper volta" OR burundi OR urundi OR "cabo verde" OR "cape verde" OR cambodia OR kampuchea OR khmer republic OR cameroon OR cameron OR cameroun OR "central african republic" OR "ubangi shari" OR chad OR chile OR china OR colombia OR comoros OR "comoro islands" OR "iles comores" OR mayotte OR "democratic republic of the congo" OR "democratic republic congo" OR congo OR zaire OR "costa rica" OR "cote dvoire" OR "cote d ivoire" OR "cote divoire" OR "cote d ivoire" OR "ivory coast" OR croatia OR cuba OR cyprus OR "czech republic" OR czechoslovakia OR djibouti OR "french somaliland" OR dominica OR "dominican republic" OR ecuador OR egypt OR "united arab republic" OR "el salvador" OR "equatorial guinea" OR "spanish guinea" OR eritrea OR estonia OR eswatini OR swaziland OR ethiopia OR fiji OR gabon OR "gabonese republic" OR gambia OR "georgia (republic)" OR georgian OR ghana OR “gold coast” OR gibraltar OR greece OR grenada OR guam OR guatemala OR guinea OR “guinea Bissau” OR guyana OR “british Guiana” OR haiti OR hispaniola OR honduras OR hungary OR india OR indonesia OR timor OR iran OR iraq OR "isle of man" OR jamaica OR jordan OR kazakhstan OR kazakh OR kenya OR "democratic peoples republic of korea" OR "republic of korea" OR "north korea" OR "south korea" OR korea OR kosovo OR kyrgyzstan OR kirghizia OR kirgizstan OR “kyrgyz republic” OR kirghiz OR laos OR lao pdr OR "lao peoples democratic republic" OR latvia OR lebanon OR "lebanese republic" OR lesotho OR basutoland OR liberia OR libya OR “libyan arab Jamahiriya” OR lithuania OR macau OR macao OR "republic of north macedonia" OR macedonia OR madagascar OR “malagasy republic” OR malawi OR nyasaland OR malaysia OR "malay federation" OR "malaya federation" OR maldives OR "indian ocean islands" OR "indian ocean" OR mali OR malta OR micronesia OR "federated states of micronesia" OR kiribati OR "marshall islands" OR nauru OR "northern mariana islands" OR palau OR tuvalu OR mauritania OR mauritius OR mexico OR moldova OR moldovian OR mongolia OR montenegro OR "montenegro republic" OR morocco OR ifni OR mozambique OR "portuguese east africa" OR myanmar OR burma OR namibia OR nepal OR "netherlands antilles" OR nicaragua OR niger OR	879,911

	nigeria OR oman OR muscat OR pakistan OR panama OR "papua new guinea" OR "new guinea" OR paraguay OR peru OR philippines OR philipines OR phillipines OR phillippines OR poland OR "polish peoples republic" OR portugal OR "portuguese republic" OR "puerto rico" OR romania OR russia OR "russian federation" OR ussr OR "soviet union" OR "union of soviet socialist republics" OR rwanda OR ruanda OR samoa OR "pacific islands" OR polynesia OR "samoan islands" OR "navigator island" OR "navigator islands" OR "sao tome and principe" OR "saudi arabia" OR senegal OR serbia OR seychelles OR "sierra leone" OR slovakia OR "slovak republic" OR slovenia OR melanesia OR "solomon island" OR "solomon islands" OR "norfolk island" OR "norfolk islands" OR somalia OR "south africa" OR "south sudan" OR "sri lanka" OR ceylon OR "saint kitts and nevis" OR "st. kitts and nevis" OR "saint lucia" OR "st. lucia" OR "saint vincent and the grenadines" OR "saint vincent" OR "st. vincent" OR grenadines OR sudan OR suriname OR surinam OR "dutch guiana" OR "netherlands guiana" OR syria OR "syrian arab republic" OR tajikistan OR tadjikistan OR tadzhikistan OR tadzhik OR tanzania OR tanganyika OR thailand OR siam OR "timor leste" OR "east timor" OR togo OR "togolese republic" OR tonga OR "trinidad and tobago" OR trinidad OR tobago OR tunisia OR "turkey (republic)" OR turkey OR turkmenistan OR turkmen OR uganda OR ukraine OR uruguay OR uzbekistan OR uzbek OR vanuatu OR "new hebrides" OR venezuela OR vietnam OR "viet nam" OR "middle east" OR "west bank" OR gaza OR palestine OR yemen OR yugoslavia OR zambia OR zimbabwe OR "northern rhodesia" OR "global south" OR "africa south of the sahara" OR "sub saharan africa" OR "subsaharan africa" OR "africa, central" OR "central africa" OR “africa, northern” OR “north Africa” OR “northern Africa” OR magreb OR maghrib OR sahara OR “africa, southern” OR “southern Africa” OR “africa, eastern” OR “east africa” OR “eastern Africa” OR “africa, western” OR “west Africa” OR “western Africa” OR “west indies” OR “indian ocean islands” OR “caribbean region” OR “caribbean islands” OR caribbean OR “central America” OR “latin America” OR "south and central america" OR “south America” OR “asia, central” OR “central asia” OR “asia, northern” OR “north asia” OR “northern asia” OR “asia, southeastern” OR “southeastern asia” OR “south eastern asia” OR “southeast asia” OR “south east asia” OR “asia, western” OR “western asia” OR “europe, eastern” OR “east Europe” OR “eastern Europe” OR “developing country” OR “developing countries” OR “developing nation? ” OR “developing population? ” OR “developing world” OR “less developed countr*” OR “less developed nation? ” OR “less developed population?” OR “less developed world” OR “lesser developed countr*” OR “lesser developed nation? ” OR “lesser developed population? ” OR “lesser developed world” OR “under developed countr*” OR “under developed nation? ” OR “under developed population? ” OR “under developed world” OR “underdeveloped countr*” OR “underdeveloped nation? ” OR “underdeveloped population? ” OR “underdeveloped world” OR “middle income countr*” OR “middle income nation?” OR “middle income population?” OR “low income countr*” OR “low income nation?” OR “low income population?” OR “lower income countr*” OR “lower income nation?” OR “lower income population?” OR “underserved countr*” OR “underserved nation?” OR “underserved population?” OR “underserved world” OR “under served countr*” OR “under served nation?” OR “under served population?” OR “under served world” OR “deprived countr*” OR	

	“deprived nation?” OR “deprived population?” OR “deprived world” OR “poor countr*” OR “poor nation?” OR “poor population?” OR “poor world” OR “poorer countr*” OR “poorer nation?” OR “poorer population?” OR “poorer world” OR “developing econom*” OR “less developed econom*” OR “lesser developed econom*” OR “under developed econom*” OR “underdeveloped econom*” OR “middle income econom*” OR “low income econom*” OR “lower income econom*” OR “low gdp” OR “low gnp” OR “low gross domestic” OR “low gross national” OR “lower gdp” OR “lower gnp” OR “lower gross domestic” OR “lower gross national” OR lmic OR lmics OR “third world” OR “lami countr*” OR “transitional countr*” OR “emerging economies” OR “emerging nation? ”):ti,ab	
#8	#6 OR #7	923,631
#9	#1 AND #4 AND #5 AND #8	934
#10	#9 AND [embase]/lim NOT [medline]/lim	237

Health Systems Evidence

No	Search terms	Hits
#1	(vaccination OR vaccines) AND (acceptance OR confidence OR uptake OR improve OR participation OR hesitancy) Filters: Document type (overviews of systematic reviews, systematic reviews of effects, systematic reviews addressing other questions); Date range (10 years)	84
#2	Included	1

Appendix S2 – List of organisations, other resource databases and unpublished resource articles that were hand searched

**Organisation and Weblink**
1. Africa CDC www.africacdc.org<br>2. China CDC www.chinacdc.cn<br>3. World health Organization www.who.int<br>4. Ministry of health and family welfare www.mohfw.gov.in<br>5. National Institute of Communicable Disease www.nicd.ac.za<br>6. Wiley Online Library https://novel-coronavirus.onlinelibrary.wiley.com/<br>7. Elsevier https://www.elsevier.com/connect/coronavirus-information-center<br>8. medRxiv and bioRxiv http://connect.medrxiv.org/relate/content/181<br>9. Oxford Academic https://academic.oup.com/journals/pages/coronavirus<br>10. JAMA Network https://jamanetwork.com/journals/jama/pages/coronavirus-alert
**Resource article and Resource link**
1. https://apic.org/professional-practice/emergency-preparedness/<br>2. http://www.nicd.ac.za/assets/files/Healthcare%20Workers%20Handbook%20on%20influenza%20in%20SA_%205%20May%202015.pdf<br>3. https://www.hpsc.ie/az/respiratory/influenza/pandemicinfluenza/guidance/pandemicinfluenzapreparednessforireland/supplement10tochapter10/File,3303,en.pdf<br>4. https://health.mo.gov/emergencies/panflu/pdf/panfluplanhealthcare.pdf<br>5. https://www.who.int/csr/disease/ebola/protective-measures-staff/en/<br>6. https://www.who.int/emergencies/diseases/novel-coronavirus-2019/technical-guidance/patient-management<br>7. https://www.who.int/docs/default-source/coronaviruse/mental-health-considerations.pdf?sfvrsn=6d3578af_10<br>8.https://apps.who.int/iris/bitstream/handle/10665/44633/9789241501866_eng_handout.pdf?sequence=3&isAllowed=y

Appendix S3 - Preferred Reporting Items for Systematic reviews and Meta-Analyses (PRISMA) Flow Chart

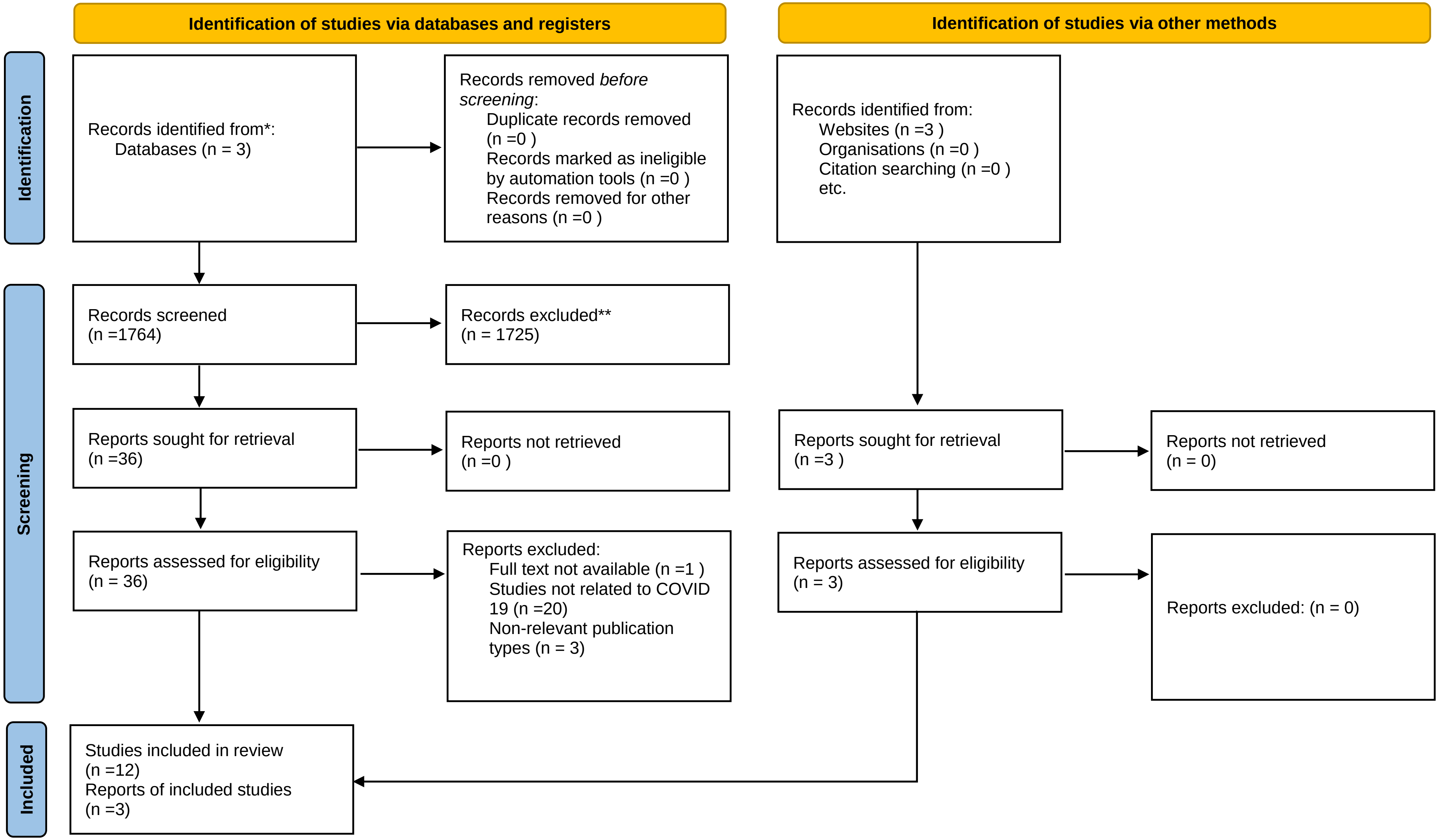

From: Page MJ, McKenzie JE, Bossuyt PM, Boutron I, Hoffmann TC, Mulrow CD, et al. The PRISMA 2020 statement: an updated guideline for reporting systematic reviews. BMJ 2021;372:n71. doi: 10.1136/bmj.n71. For more information, visit: http://www.prisma-statement.org/
